# Cognitive and Affective Learning in English as a Foreign Language/English as a Second Language Instructional-Learning Contexts: Does Teacher Immediacy Matter?

**DOI:** 10.3389/fpsyg.2021.759784

**Published:** 2021-10-13

**Authors:** Xiaoyan Wang

**Affiliations:** School of Foreign Languages, Shangqiu Normal University, Shangqiu, China

**Keywords:** affective learning, cognitive learning, instructional-learning, teacher immediacy, social behavior

## Abstract

A noteworthy frame of the literature has maintained the idea that communication in the classroom is dominant in language education, and in the process of language learning, teachers as an important figure may apply several ways to develop interpersonal relationships and social manners, such as teacher immediacy that has been established to support affective and cognitive learning in instructional settings. Therefore, this theoretical review tries to systematically refocus on the existing literature about teacher immediacy and its types, such as non-verbal and verbal, and their significant connections with affective and cognitive education. To this end, this review focuses on social behavior to review the eminence of teacher immediacy in the classroom and unquestionably exemplify their relationship with affective and cognitive learning. As a final fact, this review has been intended to consider the prevailing literature about teacher behavior, and suggestions and recommendations have been presented correspondingly for language teaching stakeholders in the educational setting.

## Introduction

Universally, teachers are noted as the main resources in any educational system, and the communication between teachers and their learners has been regarded as an essential part of the instructional cycle of language learning (Pishghadam et al., 2019; Derakhshan et al., 2020). Teachers ought to be multi-capable to support their work since expert teachers are both transferring information and preparing and arranging the exercises that would be introduced in class to get the ideal outcome (Ribahan, 2018). Among the factors related to scholastic achievement in higher education, a subset of studies inspected factors related explicitly to instructional methodologies and within the classification of instruction, results showed that the social communications of learners with teachers were more regularly connected with positive effect sizes than other factors (Schneider and Preckel, 2017; Chen and Liu, 2021). Furthermore, a vigorous issue in the success and accomplishment of learners is the communication skills of teachers (Khan et al., 2017). The art of educating is correspondence-rich, utilizing verbal, non-verbal, and composed modalities (Rosati-Peterson et al., 2021). Besides, the greater degree of the practice of verbal and non-verbal communication had a dominant function in the success of learners to a great extent (Balat et al., 2019), which is warranted as a result of the presence of emotive, instructive, sympathetic, and persistent communication between learners and teachers. Moreover, a significant segment viewed as firmly identified with the nature of schooling was the relational practices of teachers (Omar et al., 2014).

For the reason that both learners and teachers are similarly accountable for the effective acknowledgment of the instructional and learning cycles, their connection and rapport are significant (Xie and Derakhshan, 2021). Due to the prominence of teacher-learner relational connections, a great amount of consideration has been drawn to its principle and excellence (Nayernia et al., 2020; Derakhshan, 2021; Pishghadam et al., 2021). In addition, from the time of Plato and Socrates, teacher-learner association and the related results have been the focal point of many inquiries (Violanti et al., 2018; Xie and Derakhshan, 2021), and it has been fairly stated that positive teacher-learner relational connections are the solid facilitators of a wide scope of beneficial learner-related results such as commitment, learning, accomplishment, prosperity, motivation, resilience, enjoyment, achievement, and hope, among others (Aldhafiri, 2015; Derakhshan et al., 2019; Frymier et al., 2019; Derakhshan, 2021; Pishghadam et al., 2021; Wang et al., 2021).

While educational interaction is regularly comprehended across the discipline, the educational correspondence (i.e., the role of correspondence in education) is not clear all the time. The educational correspondence centers around the role of correspondence in all educating and training settings, in addition to settings across the entire life expectancy (McCroskey et al., 2006b) and settings throughout the whole life period (Nussbaum and Friedrich, 2005). Similar to how relational contact happens in personal connections in the work environment and classes, the educational relationship might occur across grade levels, educational settings, and topics (Myers, 2010).

Teacher immediacy is deemed as one of the utmost obligatory means in the path of emerging relationships, which utilizes a central part in which both teachers and students must endure to reduce the gap between them, lessen fear and pressure, and denote friendliness (LeFebvre and Allen, 2014). Possibly, the manifestation of immediacy manners permits constructive education and emerging positive interactions to be conceivable, in which both contribute to advance the full perspective of students (Nguyen, 2007). Regarding the remarkable role of immediacy in learning contexts, Witt et al. (2004) explained that verbal and non-verbal manners that teachers utilize in communications with their learners can be supposed as worthwhile subjects, and these worthwhile manners can motivate learners to be more encouraged, observant, and involved throughout the learning process (Liu, 2021).

Intentionally and unintentionally, teachers and learners send and get messages that can pass on cognitive and affective data as they cooperate in the class (Miller, 2000). Certainly, learning goals should allude to three aspects, specifically, the domain of points of view (cognitive), the domain of qualities or mentalities (affective), and the domain of abilities (Putri et al., 2018). The cognitive domain is the area that incorporates mental exercises including the cycle of acknowledgment and/or disclosure that centers on the conception, the preservation, the remembrance, and the presentation of facts (Putri et al., 2018). Similarly, it incorporates relationships between components, idea arrangement, issue disclosure, and problem-solving abilities, which thus structure novel considerations. Mental exercises identified with the cognitive domain include thinking, reasoning, admiring, and envisaging. The affective domain is the area identified with mentalities and qualities which highlight the outlook and emotional state of students toward learners and/or teachers. The mentality is one of the terms in the field of psychology which deals with insight and practice that can similarly be deciphered as a construct to permit seeing an activity. The idea of mentality itself can be observed from different related components such as mentality with character, thought processes, confidence level, and so on (Putri et al., 2018). The affective domain identifies with how an individual responds to boosts or the climate encountered to give an appraisal. Affective learning results are identified with overseeing feelings, consolation, interests, and perspectives. Affective and cognitive learning have been customarily noticed as equal learning objectives; however, recently, researchers contended that the latter is an ultimate end while the former is solely a means to the end (Zhang and Oetzel, 2006).

For those researching the role of correspondence in the education of ideal learners, understanding the emotional and social parts of class correspondence is basic. Therefore, one of the strengths of our field (i.e., English language teaching) is its consideration of the relational parts of educational correspondence (Johnson et al., 2016). Even though educational correspondence has developed as a domain of study in the course of the recent 35 years, the heft of research centers on understanding learner-teacher correspondence and connections in the class (Myers, 2010). One of the regularly examined emotion-facilitating issues is the immediacy of teachers, which brings up the opinion of physical, emotive, or emotional intimacy proven through constructive interaction manners (Enskat et al., 2017). One of the fundamental educational correspondence practices in English as a Foreign Language (EFL) classes is the immediacy of teachers (Liando, 2015) which has gained more academic consideration than most different constructs in the field of educational correspondence (Richmond et al., 2006; Zhang et al., 2007).

Immediacy has been an exploratory concept in the instructional literature and has been related to numerous preferred education consequences, containing affective learning, teacher credibility, the enthusiasm of learners, and cognitive learning (Pogue and Ahyun, 2006; Comadena et al., 2007). The verbal and/or non-verbal immediacy of teachers has been interrelated with the observed affective and/or cognitive learning of learners in the class (Witt et al., 2004). In addition, it stimulates friendliness, provokes constructive emotive reactions, and gets individuals together. There are deep-rooted inquiries that individuals are enthusiastic to be closer in immediacy to those whom they are engrossed more in instructional communications (Miller et al., 2014; Kalat et al., 2018). Teachers may employ numerous verbal or non-verbal performance systems to attain immediacy that can be observed by learners and accordingly had a confident and effective influence on their language education (Ge et al., 2019).

In addition, immediacy in the practice of teachers during teaching correspondence identifies with a positive effect and expanded cognitive learning and more positive evaluation of learners made by teachers (Bicki, 2008). It similarly motivates the uplifting perspective of learners toward teachers and school. Immediacy, along these lines, influences the learning cycle and the environment in the class. Teacher immediacy is made by moving the emphasis toward learners and can be accomplished by the utilization of both verbal and non-verbal practices (Richmond et al., 2006). Affective learning has been investigated widely as both an associate and a result of teacher immediacy as studies investigating the association between immediacy and affective learning reliably uncover a solid connection between these factors (Witt and Wheeless, 2001; Chesebro, 2003).

Besides the abovementioned clarifications demonstrating the significance of the verbal and non-verbal immediacy of teachers, numerous researchers (e.g., Sutiyatno, 2018; Violanti et al., 2018; Sheybani, 2019; Lee, 2020) have indicated the fundamental role of the instantaneous manners of teachers in EFL or English as a Second Language (ESL) contexts. It is evinced that immediacy has been significantly interrelated to student affective and cognitive erudition (Allen et al., 2006). Immediacy is also significantly linked to augmented emotive and cognitive commitment in a course, learner education approval, more constructive learner assessments of a teacher (Arbaugh, 2001; Pogue and Ahyun, 2006; Velez and Cano, 2008), condensed learner attrition degrees, and learner self-efficacy principles (Gunter, 2007). Learning researchers have premeditated immediacy in relationship with factors such as teacher integrity, intelligibility, verification (Comadena et al., 2007; Goodboy and Myers, 2008; Finn and Schrodt, 2012; Schrodt, 2013), learner communication anxiety, and learner education (Allen et al., 2006; Henning, 2012).

Based on the review of the literature and in line with the function of teacher immediacy in an instructive setting, many studies have pursued to scrutinize the relationship between these relational manners, namely, immediacy and learner-associated issues such as educational commitment, participation, willingness to take part in classes, cognitive and affective learning, fulfillment, and enthusiasm (Gholamrezaee and Ghanizadeh, 2018; Kalat et al., 2018; Pishghadam et al., 2019; Hussain et al., 2021; Zheng, 2021). As an instance, adopting a quantitative approach, Gholamrezaee and Ghanizadeh (2018) examined the association between teacher immediacy and the cognitive learning of students. To do so, three close-ended questionnaires were administered to 206 university students. Inspecting the correlation between the scales, they reported that verbal and non-verbal immediacy was associated with the cognitive learning of students. Performing Structural Equation Modeling (SEM), they also found that teacher immediacy was perceived as a strong antecedent of student cognitive learning. By the same token, Kalat et al. (2018) focused on the positive consequences of the verbal and non-verbal immediacy of teachers. To this aim, some qualitative data were collected using semi-structured interviews. The analysis of the responses of interviewees indicated that verbal and non-verbal actions that instructors employ in classroom contexts can contribute to student positive behaviors, such as learning motivation and engagement. Furthermore, Hussain et al. (2021) investigated the association between perceived teacher immediacy and student academic motivation. In doing so, three validated scales were distributed among 726 college students who were voluntarily participated in the study. The results of correlational analyses illuminated a strong connection between the variables under investigation.

Although teacher immediacy has been investigated in various settings and territories and on several interindividual and intraindividual features, as Liu (2021) noted, it appears that the immediacy of language teachers has endured an unexplored area anticipating additional investigation. Language courses are different from those courses on which preceding immediacy inquiry has been presented. Within language lessons, individuals not only come around talking about language but also employ it to generate and preserve their societal setting (Nguyen, 2007).

## Teacher Immediacy

Broadly referred to as a characterizing attribute for effective learning in both customary and online learning conditions, communication is at the core of the learning experience (Swan, 2002). Besides, it is credited as an impetus for affecting the motivation of learners, dynamic learning and contribution among learners, and the accomplishment of learning results (Du et al., 2005; Sargeant et al., 2006). The idea of immediacy, initially created by a social psychologist, Albert Mehrabian, is characterized as one of the correspondence practices that improve proximity to and non-verbal association with another (Mehrabian, 1967 cited in Velez and Cano, 2008). Initially, Mehrabian stressed non-verbal immediacy; however, he also created the scientific categorization of verbal parts later (Allen et al., 2006). Immediacy is characterized as the level of apparent physical or mental proximity between individuals (Richmond, 2002 as cited in Sheybani, 2019). Immediacy, in the field of education, has been connected to the motivational characteristic of approach-aversion in that people approach what they are interested in and stay away from what they are not attracted by (Mehrabian, 1967 as cited in Velez and Cano, 2008). In light of Mehrabian's scientific categorization of immediacy, teacher immediacy can be arranged as verbal and non-verbal practices that happen during a learner-teacher association that would develop physical and mental proximity between the two (Bozkaya and Aydin, 2007). Immediacy is a factor identified with the teaching demeanor and correspondence practice of teachers inside the educational setting. Within this system, the literature alludes to teachers or educating immediacy (Stamatis, 2014). Immediacy is a specialized instrument with an extraordinary worth that teachers possess. The educating immediacy of teachers gives learners a significant learning motivating force. The perspectives on learners about the correspondence practice of teachers and their non-verbal immediacy are connected to learning results, and learners are particularly highly motivated to learn when teachers convey the data explicitly by non-verbal immediacy and react dependably (McCroskey et al., 2006a).

Teacher immediacy is characterized as the verbal and non-verbal signals diminishing the teacher-learner physical and/or mental distance (Estepp and Roberts, 2015). As a matter of fact, teacher immediacy is a correspondence practice such as verbal and non-verbal correspondence components. Verbal immediacy alludes to elaborate contrasts in articulation, in light of which inferences are made concerning what is liked and so forth. This alludes to verbal articulations utilized by teachers. For instance, as stated by Velez and Cano (2012), a teacher could utilize the phrase “our class” and not “my class.” Verbal immediacy also incorporates verbal messages communicating sympathy, straightforwardness, generosity, reward, acclaim, sense of consideration, humor, individual information, and the readiness of teachers to include learners in correspondence (Pladevall-Ballester, 2015). Teacher immediacy works with fulfilling the necessities of learners (Frymier, 2016). Verbal immediacy practices incorporate taking part in cordial discussions with learners, getting some information about their viewpoints, and utilizing humor, while non-verbal immediacy signals involve having a casual stance, inclining forward, having fitting eye contact, and grinning at learners (Park et al., 2009; Wendt and Courduff, 2018; Derakhshan, 2021). Characterized as correspondence practices that upgrade proximity, non-verbal immediacy has been a significant domain for correspondence research for over 40 years (Pribyl et al., 2004). The non-verbal teaching immediacy is characterized as the practice that enhances proximity and non-verbal cooperation between the correspondence parties. It is the capacity of teachers to communicate sentiments, warmth, closeness, and a sense of belonging (Velez and Cano, 2012). This can be accomplished through eye contact, body position and movements, signals, grins, and expressiveness (Zhang and Sapp, 2013). The non-verbal immediacy construct depends on the possibility that the non-verbal practices of teachers advance the sensations of excitement, like, delight, and predominance. These sentiments are interceded through activities, for example, eye contact, body position, actual closeness, individual touch, and body movement to stimulate the consideration (Rocca, 2007) and interest of learners during teaching. Most non-verbal immediacy of teachers emphasizes practices, for example, eye contact, signals, body position, grinning, vocal expressiveness, movement, and locality (Liando, 2015).

A series of agencies that can ascertain distinctive behaviors such as smiling, verbal emotion, and a situation of the body has been established by immediacy scholars, and these types of immediacy variables can be taught to teachers to increase the learner-teacher rapport, student enthusiasm, commitment, and cognitive learning (Velez and Cano, 2008). Based on the studies conducted through decades about immediacy, it is generally recommended that teachers should enhance their immediacy manners for optimal success in the classroom interaction and communications through which immediate teachers build a setting where student enthusiasm and engagement can flourish, which as a result regulate their emotions (Mazer and Stowe, 2015; Greenier et al., 2021).

Non-verbal immediacy is assumed as extra-language communications guided by teachers to learners intended at creating spiritually positive interactions. They are associated with the affective realm of communication. The non-verbal clues are intermediated through such active teaching behaviors as proper eye contact, the usage of motions, movement about the class, vocal variability, and the practice of humor (Chesebro and McCroskey, 2001).

## Implications and Future Directions

This review set forth several implications in language education. Among the manners that teachers are inclined to establish in courses is verbal/non-verbal immediacy that has considerable impacts on the affective and cognitive learning of learners. The suggestions and recommendations of this study become more vivacious in assisting teachers to upturn the commitment and achievement of learners through emerging their immediacy. Successful immediacy courses provide teachers with a noble opportunity to increase more information about the prominence of communication and then encourage their verbal and non-verbal immediacy. By promoting immediacy, constructing behaviors in teachers, the learning of learners, stress tolerance, motivation, satisfaction, and self-image would expand (Chesebro and McCroskey, 2001). The satisfaction of learners is accomplished by inspiring more learner involvement and negotiation in the language class so that the learners can cultivate the abilities to talk over thoughts and make conclusions (Brookfield and Preskill, 2012) that the fulfillment of learner interactions result in upgraded learner knowledge, success, and achievement (Richmond et al., 2006). Through high verbal and non-verbal immediacy of teachers, learners were fortified to exchange their notions without distress and teachers greeted them for conversation and debate not only in the classroom but also out of the classroom. These learners felt that they were also able to increase their negotiation talents and capabilities. Teacher immediacy, affirmation, and affinity-seeking promote positive teacher-learner connections, thereby promoting fulfillment. Utilizing immediacy in-class discussion, teachers, and learners turns into better relationships and connectedness and also undergoes greater quality in communication. In particular, when learners feel associated with teachers due to these practices, they are bound to have their relatedness needs fulfilled (Frymier, 2016). The main practical contribution of this review is that if teachers are intended to upturn the satisfaction of learners of the progress, encourage them to obtain the lessons, and expand their interaction, engagement, and cognitive learning, they should emphasize increasing the immediacy of teachers. Teachers can apply verbal immediacy manners, such as humor, commitment in dialogs with learners prior, afterward, or outdoors, inspiring learners to speak, demanding involvement, calling them by name, admiring their effort, and being accessible for learners outdoors if they have any problems.

The review serves to show support for teacher immediacy as an important element of learner success, that is, the goal of language learning. Immediacy in the class assists learners to form a constructive opinion of the ability, credibility, and thoughtful approach of teachers (Rocca, 2007; Gao, 2021; Liu, 2021). These positive insights can also support to boost learner involvement in the class, and both types of immediacy, namely, verbal and non-verbal communication, are significant (Woods and Baker, 2004) while the former helps to convey subject matter, and the latter is appropriate in increasing teacher-learner relationships (Richmond et al., 2006). Immediacy in a class can also embolden the progress of relational fascination between teachers and learners (Sidelinger and Booth-Butterfield, 2010). Through immediacy, the students become aware that their teachers care about them that consequently boosts their communication in the class (Comadena et al., 2007). Instructive academics (Henson and Denker, 2009) proposed that teachers must pay attention to each student, and this helps them to realize the teaching objective. Teachers taking them into account motivate them to take part and act together in the classroom.

Based on the review, it was proved that more learner awareness of immediacy causes more motivation in learners and that better learner motivation brings about more view of cognitive learning, besides higher effective ones. The role of teacher immediacy behaviors is assured in constructing cognitive learning (Frymier and Houser, 2000) as the immediacy of a teacher aids to eradicate the physical or mental space between the students which builds an insight that the teacher is close to them and this principle reintroduces the teacher-learner connection, which is considered as a prompting aspect of cognitive learning. Thus, the immediacy of teachers acts as a facilitator that moderates the apparent gap between teachers and learners and increases their language teaching (Allen et al., 2006).

In addition, when a teacher is highly immediate, it is obvious that they have a positively better influence on their learner motivation, which refers to the affective learning that can be defined as the encouraging principles that learners stick on to the behaviors of teachers in the classroom and it is proved that there is a positive relationship between the immediacy and affective learning of teachers (Frymier and Houser, 2000; Allen et al., 2006; Pogue and Ahyun, 2006). It surges motivation in the progression of education that may adjust the manners of learners. Teachers with immediate behavior are deemed more communicative that can govern their class (Mottet et al., 2006). The verbal and non-verbal immediacy of teachers leads to constructive social interactions with learners, which pinpointed that learners are less worried and more self-initiated in the instructional process in which they express that the inspiration and enthusiasm of students for education are augmented (Allen et al., 2006). An important implication taken from this review is that teachers should be aware that their immediacy, either verbal or non-verbal, successfully and strongly boosts the enthusiasm of learners for language learning. Therefore, on the one hand, teachers should be more thoughtful in their classes, keeping in mind that their immediacy could have a positive effect, increasing the motivation of learners. On the other hand, the less immediacy of teachers decreases and even diminished the interests and affective learning of learners (Pogue and Ahyun, 2006).

Similarly, this review is beneficial to those supervisors and administrators who wish to select the best teachers for their language school or institute since the information of teachers, classroom administration capability, and other important teacher features; for example, the capability to create an immediate rapport with learners should be deliberated as a new prominent issue. Furthermore, aiming to aid learners struggling with negative issues such as stress, boredom, burnout, low self-confidence, or other factors (Seifalian and Derakhshan, 2018; Fathi and Derakhshan, 2019; Derakhshan et al., 2021; Zawodniak et al., 2021), language faculty should certainly decide on instructors with great verbal/non-verbal immediacy. Students are proficient enough to deal better with difficulties and problems in class when they feel enthusiastically close enough to their teacher so they can control and adjust demanding circumstances and opposing occasions in a mode that does not offend them in educational contexts.

As the purpose of education is to look for routes to encourage students, possibly teacher training teachers should study the upshot of immediacy, so all the stakeholders of learning progression should be stimulated to assess and contemplate their verbal and non-verbal communication approaches. If teachers propose to assist an ideal classroom situation, they must direct caring and considerate communication messages to all learners. Training of teachers is becoming gradually mutual in different contexts (Gibbs and Coffey, 2004) so it is imperative to develop the understanding of teachers about both the principle and training of immediacy-constructing manners with the aim of heightening learner education. Therefore, teachers are supposed to take part in some seminars that concentrate on the social side of the classroom milieu to study detailed and precise approaches and tactics that further lead to positive interactions among learners. Due to the great role of verbal/non-verbal immediacy in education and their distinctive effect on the success and feelings of learners (Wei and Wang, 2010), it is suggested that teaching policymakers and experts put emphasis on both types of immediacy by presenting teacher immediacy instructing progressions.

Some lacunas were also found in the existing literature, which needs to be highlighted. First and foremost, there is a dearth of research to investigate in what way the social interaction practices of teachers can be heightened and as it is concluded that based on the review of the related literature, teacher immediacy is deemed to be a crucial element, so future studies, particularly experimental ones, should be carried out to present instructional treatment to a group of teachers on a specific facet of relational interactions to notice how receiving intervention can encourage the social behavior of teachers. Second, most of the previous studies in this area were carried out in general education (Bozkaya and Aydin, 2007; Estepp and Roberts, 2015; Enskat et al., 2017). That is, the consequences of immediate behaviors for language learners, such as EFL and ESL students, received scant attention. Thus, to narrow this gap in the literature, future inquiries are required to delve into the impact of the verbal and non-verbal immediacy of teachers on the academic behaviors of EFL and ESL students. Third, the majority of existing studies were purely quantitative, using close-ended questionnaires to gather the data. To attain more comprehensive findings, future investigations are recommended to triangulate the data employing other data collection instruments, such as observations, structured/semi-structured interviews, and diary/narrative writings. Finally, contextual factors, such as age, gender, and educational background, were not among the main concerns of previous studies. To identify to what extent age, gender, and educational background of participants can alter their perceptions regarding the effects of teacher interpersonal factors, notably verbal and non-verbal immediacy, future studies should measure the probable effects of such variables.

## Author Contributions

The author confirms being the sole contributor of this work and has approved it for publication.

## Conflict of Interest

The author declares that the research was conducted in the absence of any commercial or financial relationships that could be construed as a potential conflict of interest.

## Publisher's Note

All claims expressed in this article are solely those of the authors and do not necessarily represent those of their affiliated organizations, or those of the publisher, the editors and the reviewers. Any product that may be evaluated in this article, or claim that may be made by its manufacturer, is not guaranteed or endorsed by the publisher.
